# Determinants of late disease-stage presentation at diagnosis of HIV infection in Venezuela: A case-case comparison

**DOI:** 10.1186/1742-6405-5-6

**Published:** 2008-04-16

**Authors:** Maeva A Bonjour, Morelba Montagne, Martha Zambrano, Gloria Molina, Catherine Lippuner, Francis G Wadskier, Milvida Castrillo, Renzo N Incani, Adriana Tami

**Affiliations:** 1 Department of Biomedical Research, Royal Tropical Institute, Amsterdam, The Netherlands; 2 Department of Epidemiology and Biostatistics, Radboud University Nijmegen Medical Centre, Nijmegen, The Netherlands; 3 Centre for Integral Attention for Sexually Transmitted Diseases and HIV/AIDS, National Program of HIV/AIDS, Ministry of Health and Social Development, Valencia, Venezuela; 4 Department of Biology and Society, Faculty of Earth and Life Sciences, Free University of Amsterdam, Amsterdam, The Netherlands; 5 Department of Parasitology, Faculty of Health Sciences, University of Carabobo, Valencia, Venezuela; 6 Centre of Information Technology, Communication and Assisted Education, Faculty of Health Sciences, University of Carabobo, Valencia, Venezuela

## Abstract

**Background:**

Although Venezuela has a National Human Immunodeficiency Virus (HIV) Program offering free diagnosis and treatment, 41% of patients present for diagnosis at a later disease-stage, indicating that access to care may still be limited. Our study aimed to identify factors influencing delay in presenting for HIV-diagnosis using a case-case comparison. A cross-sectional survey was performed at the Regional HIV Reference Centre (CAI), Carabobo Region, Venezuela. Between May 2005 and October 2006 225 patients diagnosed with HIV at CAI were included and demographic, behavioural and medical characteristics collected from medical files. Socio-economic and behavioural factors were obtained from 129 eligible subjects through interviews. "Late presentation" at diagnosis was defined as patients classified with disease-stage B or C according to the 1993 Centers for Disease Control and Prevention (Atlanta, USA) classification, and "early presentation" defined as diagnosis in disease-stage A.

**Results:**

Of 225 subjects, 91 (40%) were defined as late presenters. A similar proportion (51/129) was obtained in the interviewed sub-sample. Older age (>30 years), male heterosexuality, lower socio-economic status, perceiving ones partner to be faithful and living ≥ 25 km from the CAI were positively associated with late diagnosis in a multivariate model. Females were less likely to present late than heterosexual males (odds ratio = 0.23, *P *= 0.06). The main barriers to HIV testing were low knowledge of HIV/AIDS, lack of awareness of the free HIV program, lack of perceived risk of HIV-infection, fear for HIV-related stigma, fear for lack of confidentiality at testing site and logistic barriers.

**Conclusion:**

Despite the free Venezuelan HIV Program, poverty and barriers related to lack of knowledge and awareness of both HIV and the Program itself were important determinants in late presentation at HIV diagnosis. This study also indicates that women; heterosexual, bisexual and homosexual men might have different pathways to testing and different factors related to late presentation in each subgroup. Efforts must be directed to i) increase awareness of HIV/AIDS and the Program and ii) the identification of specific factors associated with delay in HIV diagnosis per subgroup, to help develop targeted public health interventions improving early diagnosis and prognosis of people living with HIV/AIDS in Venezuela and elsewhere.

## Background

With an estimated 110,000 people living with Human Immunodeficiency Virus (HIV)/Acquired Immune Deficiency Syndrome (AIDS) (PLWHA) in 2005, Venezuela is among the countries with the highest HIV prevalence (0.7% in adults) in Latin America [1]. The ratio men to women gradually changed from 19:1 in the eighties to 2:1 in 2004 [2]. As in the rest of Latin America, HIV is mostly spread through sexual transmission, accounting for 90% of all reported HIV-infections between 1982 and 1999 [3]. Of the reported sexual transmissions of HIV 65% in that period involved men who had sex with men [3]. However, as the epidemic matures the proportion of infected heterosexual men and women is rising [2]. Analyses of data collected from 1999 to 2004 in Carabobo State showed that heterosexual transmission occurred in 61% of the cases [4].

Since 1999, the Venezuelan National HIV/AIDS Program (PNSIDA in Spanish) provides free comprehensive care for PLWHA, including diagnosis and monitoring, antiretroviral therapy (ART), treatment of opportunistic infections and other sexually transmitted infections (STIs), and prevention of mother-to-child transmission [5]. In 2005, almost 16,000 PLWHA received free ART [2]. However, of those estimated to require treatment in Venezuela in 2005, 16% did not receive it [6]. A recent study in Carabobo State found that 41% (196/491) of the HIV-infected patients attending the PNSIDA between 1999–2004 presented for diagnosis at a later disease stage [4]. This indicates that there are other factors hindering access to HIV-care than cost of diagnosis and treatment.

Early diagnosis of HIV-infection has benefits for the patient, public health and the society as a whole. Patients diagnosed at a late stage have poorer prognosis [7], whereas when started early, ART is more effective [8-11] and with early diagnosis psychosocial aspects can be better dealt with [12]. Early diagnosis also reduces HIV-transmission through clinical and behavioural preventive measures [13,14]. Finally, the early detection of HIV-infection has proven to be economically beneficial [15,16] and to improve healthcare system planning capabilities [17].

Few studies have focused on these issues in Latin America [18,19]. A high proportion of individuals in Venezuela discover they are HIV-infected too late to fully benefit from ART. However, little research has been performed on the impact of government HIV programmes and the knowledge and behaviour of the targeted populations [20]. Here we report the identification of factors associated with late presentation at HIV-diagnosis concomitantly with perceived barriers to testing in Venezuela. We furthermore highlight the importance of understanding region-specific determinants in order to improve the impact of free HIV-programs.

## Results

Between the 1st of May 2005 and the 31st of October 2006, 226 individuals were newly diagnosed with HIV at the Reference Centre for Sexually Transmitted Infections and HIV/AIDS (CAI, in Spanish) in Valencia, Carabobo region, Venezuela. One individual was excluded from the study as the patient's medical file could not be located. The outcome of interest, 'late presentation' (disease stage B or C at HIV-diagnosis [21]), occurred in 40% (91/225) of the individuals in agreement with a previous study [4].

Of the 225 included individuals, 129 (57%) were interviewed between the 25^th ^of April and the 25^th ^of October 2006. Of the 96 remaining eligible subjects one died, a second moved away, a third could not answer the questionnaire and three refused to participate; a further 90 were not interviewed either because they never attended the clinic during the study period, or because the interviewers were not available when they did. The average time between HIV diagnosis and interview was 4 months. Data collected from the patients' medical files was used to describe the total study population (n = 225). To test how representative the interviewed sample was, possible differences between the interviewed (n = 129) and non-interviewed individuals (n = 96) were examined by comparing the distribution of age, sex, marital status, education level, occupation, sexual orientation, HIV disease-stage classification [21], CD4^+ ^count, number of casual partners, condom and alcohol use and drug abuse between the two groups at the moment of HIV diagnosis (data not shown). There were no statistically significant differences except for sexual orientation, where a lower proportion of male heterosexuals was interviewed (26% vs. 47%; *P *= 0.001).

### Demographic, socio-economic and behavioural factors

The mean age was 33 years (range 15–79 years) with the majority (67%) of individuals between 20 and 40 years old and a male/female ratio of nearly 4:1 (Table 1). Most of the single (111/132) and married persons (11/15) were men, while half (32/60) of the unmarried people living with a partner were women. Only 3 females self-identified as homo- or bisexual. Bi- and homosexuals were more likely to have finished secondary school than heterosexuals (70% vs. 33%; P < 0.001).

**Table 1:** Demographic and socio-economic factors associated with late presentation at HIV diagnosis in Venezuela, Carabobo State.

	**Late presenters**		**Total**		
			
	**n**	**%**	**n**	OR*(95%CI)	***P*-value (*P*^T^)**
**SOCIO-DEMOGRAPHIC**					
Sex^a, †^					
Male	72	44.2	163	1	-
Female	19	30.6	62	0.57 (0.30–1.10)	0.094
Age (years)^a,‡^					
< 20	3	17.6	17	1	- (0.003)
20–29	24	27.0	89	1.74 (0.46–6.64)	0.417
30–39	34	55.7	61	6.02 (1.56–23.30)	0.009
>40	30	51.7	58	4.86 (1.25–18.84)	0.022
Marital Status^a ^(n = 224)					
Single	54	40.9	132	1	-
Married	9	47.4	19	1.06 (0.38–2.95)	0.912
Divorced	6	75.0	8	3.06 (0.56–16.77)	0.198
Widowed	2	40.0	5	0.69 (0.11–4.48)	0.693
Living together	20	33.3	60	0.86 (0.42–1.74)	0.670
Children^a ^(n = 219)					
0	35	32.1	109	1	-
≥ 1	52	47.3	110	2.06 (1.11–3.83)	0.022
Sexual orientation^a^					
Heterosexual	60	43.5	138	1	-
Bisexual	17	47.2	36	0.76 (0.33–1.71)	0.503
Homosexual	14	27.5	51	0.40 (0.18–0.87)	0.020
Education level^a^					
Not finished secondary school	55	46.6	118	1	-
Secondary school and higher	36	33.6	107	0.57 (0.32–1.01)	0.053
**SOCIO-ECONOMIC**					
Type of occupation^a ^(n = 223)					
Unemployed	8	42.1	19	1	-
Domestic worker	11	35.5	31	2.35 (0.54–10.28)	0.258
Manual worker	25	56.8	44	1.73 (0.55–5.46)	0.347
Self-employed/Commerce	22	50.0	44	1.41 (0.45–4.44)	0.555
Paid employee/Office worker	14	27.5	51	0.57 (0.18–1.79)	0.334
Professional/University staff	4	33.3	12	0.53 (0.11–2.55)	0.426
Student	7	31.8	22	1.38 (0.35–5.52)	0.646
Area of residence^b^					
Rural	8	57.1	14	1	-
Urban	43	37.4	115	0.34 (0.10–1.15)	0.082
Ownership residence^b^					
Owning	37	50.0	74	1	- (0.008)
Renting	8	26.7	30	0.30 (0.11–0.81)	0.017
Borrow/lodged	6	24.0	25	0.38 (0.13–1.10)	0.074
Socio-economic status^b,§^					
Low	32	50.0	64	1	
High	19	29.2	65	0.24 (0.10–0.57)	0.001

^a^Data source: patient files (n = 225). ^b^Data source: questionnaires (n = 129). Missing values are deducted by subtracting the total of individuals for each variable to the corresponding 225 (a) or 129 (b) patients. If totals are not indicated for a variable, it has no missing values. *Adjusted for age group and sex. ^†^Odds ratio only adjusted for age group. ^‡^Odds ratio only adjusted for sex. ^§^Socio-economic status was calculated for all interviewed persons as described in Methods. OR, odds ratio; CI, confidence interval; *P*^T^, Mantel-Haenszel Score test for trend *P*-value.

Older age (≥ 30 years), having children and lower education level showed a significant positive association with late presentation for HIV-testing (Table 1). Women were almost half as likely to present late as men, while homosexuals were less likely to present late than heterosexuals. Although socio-economic factors did not show a clear association except for ownership of residence, the compound variable "Socio-economic status" (SES, see Methods) indicated that individuals with lower SES were more likely to be late presenters at HIV-diagnosis (Table 1).

Late presentation was not associated with alcohol consumption, drug abuse or condom use. The proportion of late presenters was lower among those having a steady partner, however this effect was mostly found for those who knew their steady partner was HIV-infected (Table 2). Moreover, perceiving their steady partner to be unfaithful, which could be a proxy for risk perception, showed a negative association with late presentation. There was an increased trend to present late the longer a person had a steady partner (Table 2).

**Table 2:** Behavioural characteristics and knowledge attributes associated with late presentation at HIV diagnosis in Venezuela, Carabobo State.

	**Late presenters**		**Total**		
			
	**n**	**%**	**n**	OR*(95%CI)	***P*-value (*P*^T^)**
**BEHAVIOURAL CHARACTERISTICS**					
Alcohol use^a ^(n = 221)					
No alcohol	32	47.1	68	1	-
Social drinker	33	34.0	97	0.49 (0.25–0.97)	0.041
Moderate drinker	18	39.1	46	0.55 (0.24–1.25)	0.155
Alcoholic	7	70.0	10	1.61 (0.35–7.44)	0.541
Drug abuse^a ^(n = 215)					
No	82	42.1	195	1	-
Yes	8	40.0	20	0.91 (0.34–2.44)	0.855
Lifetime casual partners^b ^(n = 114)					
0	8	28.6	28	1	- (0.286)
1–10	16	34.8	46	1.66 (0.40–6.97)	0.489
>10	21	47.5	40	2.72 (0.60–12.48)	0.197
Steady partner^a ^(n = 219)					
No	52	48.6	107	1	- (0.021)
Yes, partner HIV^- ^or unknown HIV status	25	40.3	62	0.66 (0.16–2.65)	0.558
Yes, partner HIV^+^	12	24.0	50	0.42 (0.19–0.92)	0.030
Perception faithfulness steady partner^b^					
Faithful	23	56.1	41	1	-
Unfaithful/Doubting faithfulness	4	16.0	25	0.18 (0.05–0.66)	0.010
No steady partner	24	38.1	63	0.49 (0.21–1.12)	0.094
Time with steady partner (months)^a, † ^(n = 106)					
<24	10	20.8	48	1	- (0.010)
25–120	18	40.0	45	2.49 (0.95–6.52)	0.063
>120	7	53.8	13	3.01(0.75–12.15)	0.121
Condom use^a ^(n = 169)					
Never	39	38.2	102	1	-
Sometimes	13	36.1	36	0.95 (0.40–2.26)	0.911
Often	7	38.9	18	0.96 (0.32–2.94)	0.946
Always	6	46.2	13	0.71 (0.21–2.41)	0.583
Contact with commercial sex workers^b,‡ ^(n = 93)					
No	22	34.4	64	1	-
Yes	18	62.1	29	2.54 (0.99–6.54)	0.054
**KNOWLEDGE ATTRIBUTES**					
Knowledge-HIV-transmission score^b,§^					
0 = no knowledge	7	63.6	11	1	- (0.033)
1–8 = poor knowledge	9	56.3	16	0.94 (0.18–5.03)	0.944
9–15 = good knowledge	35	34.3	102	0.32 (0.08–1.26)	0.103
Awareness HIV test^b^					
Not aware of existence	9	64.3	14	1	- (0.089)
Aware of existence, but not aware it was for free	26	38.2	68	0.39 (0.11–1.38)	0.143
Aware of existence and that it was for free	16	34.0	47	0.31 (0.08–1.14)	0.078
Awareness treatment^b^					
Not aware of existence	24	40.0	60	1	-
Aware of existence, but not aware it was for free	15	34.1	44	0.79 (0.34–1.84)	0.580
Aware of existence and that it was for free	12	48.0	25	1.03 (0.37–2.86)	0.951
Awareness PNSIDA score^b, ^** (n = 128)					
0 = no awareness	7	63.6	11	1	- (0.055)
1–4 = some awareness	37	38.5	96	0.32 (0.08–1.29)	0.109
5–7 = good awareness	7	33.3	21	0.20 (0.04–1.05)	0.057
Total-HIV-knowledge score^b,†† ^(n = 128)					
0–14 = low overall knowledge	20	51.3	39	1	-
15–28 = high overall knowledge	31	34.8	89	0.51 (0.23–1.13)	0.096

^a^Data source: patient files (n = 225). ^b^Data source: questionnaires (n = 129). Missing values are deducted by subtracting the total of individuals for each variable to the corresponding 225 (a) or 129 (b) patients. If totals are not indicated for a variable, it has no missing values. *Adjusted for age group and sex. ^†^Only those with steady partner were included (n = 112). ^‡^Only men were included (n = 94). ^§^Calculated from a 15-item HIV transmission question. **Calculated by adding all awareness variables. ^††^Calculated by adding knowledge HIV transmission score, awareness PNSIDA score and one point for each correct answer to 6 true-or-false statements about HIV/AIDS. OR, odds ratio; CI, confidence interval; *P*^T^, Mantel-Haenszel Score test for trend *P*-value.

### Knowledge of HIV/AIDS

The majority of interviewed people (125/129) indicated they had heard about HIV. The main sources of information were the media, family/friends and school. Most people (118/129) said they knew how HIV was transmitted. Awareness of the existence of an HIV control program was low. Most people knew that an HIV-test existed but 59% (68/115) of these were not aware that the test was freely available (Table 2). Among the latter, 53% did not know how much a test would cost. Fewer people knew that treatment existed and only 25 knew it was available for free (Table 2).

Individuals who had never heard of HIV were more likely to be late at diagnosis than those who had (50% vs. 39%), but this effect was not significant (*P *= 0.662), possibly due to small sample size in the first group (n = 4, data not shown). Having heard about HIV at school decreased the likelihood of late presentation (OR, 0.39; 95% confidence interval (CI), 0.15–1.01), while none of the other sources of information showed any effect (data not shown). There was a decreasing trend for late presentation with increasing knowledge of HIV-transmission and awareness of the PNSIDA (Table 2). Awareness of existence and free availability of HIV testing was negatively associated with late presentation while no association was found for awareness of treatment availability. Persons with a low total-HIV-knowledge score were twice as likely to present late (*P *= 0.096, Table 2).

### Risk perception and barriers and facilitators for testing

More than half of the interviewees had felt at risk of HIV-infection before diagnosis (Table 3). The main reasons mentioned for this risk perception were having unprotected sex (n = 21), having many sexual partners (n = 21), having homosexual partners (n = 10), having an unfaithful partner (n = 7), and having an HIV-positive partner (n = 7). The main reasons mentioned for not feeling at risk were having a steady partner (n = 25), not being aware of their own risk behaviour (n = 18), not knowing about HIV (n = 10), having protected sexual intercourse (n = 8), and not having any symptoms (n = 7). The time span people felt at risk before HIV-diagnosis ranged from 1 month to 12 years, with a geometric mean of 10 months. Of those who felt at risk, almost half (31/67) indicated no health-seeking behaviour, 16 (24%) started protecting themselves or turned to family, friends or their partner for advice, and 18 (27%) went to a health centre or the CAI. The majority of the interviewed persons (71/129) indicated to have perceived no barriers to HIV-testing. This may in part be explained by lack of perception of risk for HIV-infection, since those who had felt at risk were 7 times more likely to have mentioned any barriers (*P *< 0.001). Fourteen individuals (11%) mentioned at least one of the barriers categorized under 'confidentiality testing site,' while 32 individuals (25%) mentioned barriers from the category 'fear for stigma' and 12 (9%) mentioned items indicating logistical barriers (see Methods for definitions of categories).

**Table 3:** Risk perception, barriers to testing and final model of factors independently associated with late presentation at HIV diagnosis in Venezuela, Carabobo State.

	**Late presenters**		**Total**		
			
	**n**	**%**	**n**	OR* (95%CI)	***P*-value**
**PERCEPTION OF RISK**					
Felt at risk of HIV infection (asked directly)^b^					
No	26	41.9	62	1	-
Yes	25	37.3	67	0.83 (0.39–1.79)	0.638
No perception of risk (mentioned as barrier)^b ^(n = 122)					
Not mentioned	36	35.0	103	1	-
Mentioned	12	63.2	19	4.33 (1.40–13.33)	0.011
Health-seeking behaviour when felt at risk^b,† ^(n = 65)					
No health-seeking behaviour	14	45.2	31	1	-
Protect oneself or seek advice family/friends/partner	6	37.5	16	0.52 (0.13–2.05)	0.347
Seek advice health centre/CAI	3	16.7	18	0.19 (0.04–0.88)	0.034
**BARRIERS TO TESTING**					
Confidentiality testing site^b,‡ ^(n = 117)					
Not mentioned	39	37.9	103	1	-
Mentioned	8	57.1	14	2.30 (0.71–7.50)	0.167
Fear for stigma^b,§ ^(n = 125)					
Not mentioned	35	37.6	93	1	-
Mentioned	14	43.8	32	1.41 (0.60–3.33)	0.434
Logistic constraints^b, ^** (n = 119)					
Not mentioned	39	36.4	107	1	-
Mentioned	8	66.7	12	3.95 (1.05–14.81)	0.042
Having no signs or symptoms^b^					
Not mentioned	38	33.6	113	1	-
Mentioned	13	81.3	16	4.33 (1.40–13.33)	0.011
Not-wanting-to-know HIV-status^b ^(n = 127)					
Not mentioned	36	35.0	103	1	-
Mentioned	14	58.3	24	2.53 (0.93–6.86)	0.069
Distance to CAI^a^					
≤ 25 km	71	37.0	192	1	-
> 25 km	20	60.6	33	3.15 (1.39–7.14)	0.006
**Final model of factors independently associated with late presentation at HIV diagnosis (n = 123/129)**					
**Factors**				**OR^†† ^(95% CI)**	***P*-value**
Age					
<30 years				1	-
≥ 30 years				5.34 (1.70–16.76)	0.004
Sexuality					
Male heterosexual				1	-
Male homosexual				0.22 (0.05–0.92)	0.039
Male bisexual				2.38 (0.46–12.41)	0.302
Female				0.23 (0.05–1.06)	0.059
Perception faithfulness partner					
Faithful				1	-
Unfaithful/Doubting faithfulness				0.078 (0.01–0.56)	0.011
Distance to CAI					
<25 km				1	-
≥ 25 km				16.69 (3.02–92.11)	0.001

^a^Data source: patient files (n = 225). ^b^Data source: questionnaires (n = 129). Missing values are deducted by subtracting the total of individuals for each variable to the corresponding 225 (a) or 129 (b) patients. If totals are not indicated for a variable, it has no missing values. *Adjusted for age group and sex. ^†^Only those who indicated to feel at risk of HIV infection were included (n = 67). ^‡^Set as 'mentioned' if: confidentiality test, doubt correctness result, attitude personnel or being seen at site was mentioned or agreed. ^§^Set as 'mentioned' if: fear of loosing partner/family/job/children or fear for rejection was mentioned or agreed. **Set as 'mentioned' if: no time, inconvenient location, no transport money, costs treatment or costs test was mentioned or agreed. ^††^Adjusted for SES, having an HIV^+ ^partner, total-HIV-knowledge score, testing as part of screening and the other variables in this model. OR, odds ratio; CI, confidence interval.

Although not significant, late presentation was slightly higher among those that had not felt at risk of HIV-infection than those who did when the question "did you feel at risk" was asked directly. However, mentioning not to have perceived themselves to be at risk as *a barrier *to HIV-testing showed a strong association with late presentation, even after adjusting for age group and sex (Table 3). People who had perceived barriers to HIV-testing were more likely to present late but this effect was not significant (*P *= 0.344). For the categories of barriers 'fear for stigma' and 'confidentiality testing site' a similar non significant association was found. Persons indicating logistical constrains were almost 4 times more likely to present late (*P *= 0.042; Table 3). Mentioning not-wanting-to-know their HIV status was associated with late presentation (Table 3), while mentioning fear to be diagnosed positive was not (OR, 1.00; 95%CI 0.39–2.59), indicating that this might have a bi-directional effect on testing behaviour. Of the 13 persons that presented late and mentioned not-having-symptoms-yet as a barrier, 9 (69%) had felt at risk, indicating that feeling healthy might prevent people from converting their perception of risk into the act of HIV-testing. Persons living ≥ 25 km away from the CAI were 3 times more likely to present late than those who did not(Table 3). However, reported time and transport costs to CAI were not associated with late presentation.

**Figure 1:**
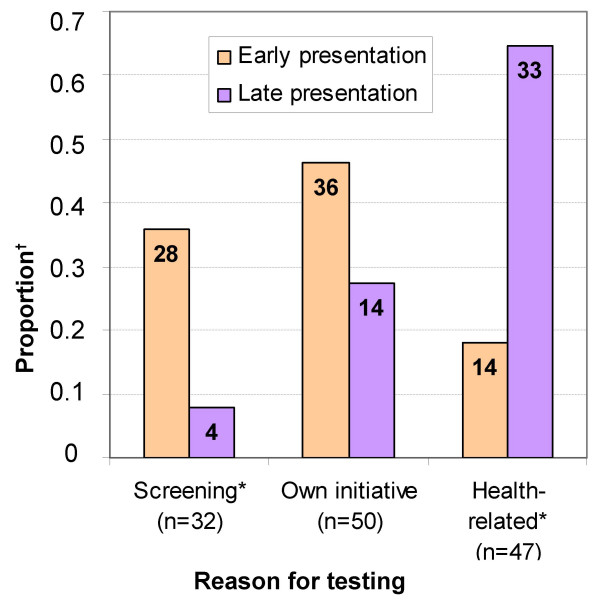
**Proportion of early and late presenters, by reason for testing**. *Screening *consisted of screening at blood bank, antenatal and pre-surgery screening and screening as part of health certification; *Own initiative *consisted of testing because of curiosity, feeling at risk of HIV-infection, having had STIs, many sexual partners, unprotected sex, an unfaithful partner, or testing on advice of partner, family or friends; *Health-related *consisted of referral by a health centre, the respondent or partner showing HIV-related symptoms and having HIV-infected partners or children. *P <0.05. ^†^Number of individuals is noted within each bar.

Taking the HIV-test on their own initiative (50/129) or for health-related reasons (47/129) were mentioned by most individuals, while the remaining 32 individuals mentioned screening as the reason for testing. Testing on own initiative was negatively associated with late presentation (OR, 0.44; CI, 0.21–0.94), while testing for health-related reasons increased the likelihood of being late 8 times (*P *< 0.001, Figure 1). Of those tested as part of screening, 13% was still diagnosed in a late stage of HIV-infection.

### Multivariate analysis

For a final model, sexual orientation and sex were combined into one variable ('sexuality') with women, heterosexual men, homosexual men and bisexual men as the four categories. Persons living <25 km away from the CAI, of younger age, that did not perceive their partner to be faithful and women and homosexual men remained less likely to present late after adjusting for SES, having an HIV^+ ^partner, overall HIV knowledge, and screening as reason for testing (Table 3).

## Discussion

This study is, to our knowledge, the first in Latin America to have explored factors associated with late presentation at HIV-diagnosis concomitantly with perceived barriers to testing. Only two other studies have been performed in Latin America; a study in French Guiana examined determinants of late HIV-diagnosis [19] and another in Brazil looked at barriers to testing during antenatal care [18]. In developed and Sub-Saharan African countries, most studies either focus on perceived barriers and attitudes to voluntary testing [22-25] or on determinants of late presentation for HIV-testing [10,26-29]. Few studies have actually examined the pathway – and hurdles – of those who present late for HIV-diagnosis, and most of them were carried out in developed countries [30-32]. Using a case-case comparison this study has identified factors involved with late presentation for HIV-diagnosis within a free HIV-program in Latin America. In line with other studies examining HIV-testing behaviour and late presentation, we have found that older age [7,10,19,26,33], lower educational level [18,27], lower SES [28] and heterosexual orientation in men [10,12,28] increase the likelihood of late presentation. Moreover, lack of knowledge about HIV/AIDS [34], lack of awareness about the free services provided by the PNSIDA, lack of perceived risk of infection [23,24,28,35], psychological barriers [23,25,28,34-36] and logistical constraints [23,24,36] are associated with this delay in HIV-testing.

Since it is difficult to determine the moment of infection, low CD4^+^-cell count at diagnosis [19,26,29,30] or rapid progression to AIDS [10,27,28,31,37] have been used to define late presentation. In contrast, we used the CDC classification system for HIV-infection [21] encompassing the whole clinical picture at the moment of diagnosis which allowed the inclusion of all individuals newly diagnosed within the period of study. Our case definition was deliberately chosen to avoid ascertainment bias in our study population since around 60% of individuals do not have a CD4 count up to at least three months after HIV diagnosis [4]. Moreover, differential distribution of individuals without CD4 counts introduces further bias, as the majority of patients without CD4 counts represent disease-stage A patients. A limitation of our study is that only 57% of the study population could be interviewed. These individuals had a lower proportion of male heterosexuals than non-interviewed. Heterosexual men may be more reluctant to be interviewed than bisexual or homosexual men especially if the latter feel supported by dedicated NGOs making them more open to discuss their HIV status. Other possible limitations refer to recall bias as most questions related to the time before or at diagnosis, and bias due to the setting of the interview, since respondents might have been reluctant to mention barriers related to the CAI when the interview was conducted by the clinic's staff. However, we tried to minimise these by proper training of interviewers and by ascertaining that the interviewees' answers referred to the appropriate time before or at diagnosis. The use of a case-case comparison minimises differential recall bias that may occur in case-control studies [38]. The CAI is the reference centre for the regional PNSIDA but it is possible that very ill patients may be admitted directly to tertiary hospitals. In this case, these patients are either reported to CAI after HIV diagnosis or, more commonly, blood samples are sent to CAI for diagnosis. If any of these patients were diagnosed within the period of our study they were also included in the sampled population.

Delayed HIV diagnosis has been related with age in most studies. While some find older age influencing late presentation (this study, [7,10,19,26,33]) others find younger individuals more at risk of a late diagnosis [27,37]. Study design may have influenced this contrasting association with age where exclusion criteria may have limited how representative the study sample was as previously noted by other authors [27,28]. Older individuals in Venezuela may be less aware of HIV and more reluctant to come forward to HIV testing compared to younger individuals.

In our study, risk perception measured by different proxies showed contrasting associations with late presentation. Many studies have identified risk perception as a motivator for HIV-testing [26,30,39]. However, it was also found that for some people, risk perception acts as a deterrent for HIV-testing [17,28]. This bi-directional effect might have distorted some of the associations with late presentation in our study. For instance, when risk perception was asked about directly, no association could be found, while mentioning not-wanting-to-know their HIV status as a barrier showed a positive association and mentioning fear to be diagnosed positive or perceiving their partner to be unfaithful a negative association with late presentation at diagnosis. Not-wanting-to-know their HIV-status could be related to fear for HIV-related stigma as well as to general coping strategies to deal with a possible diagnosis of a life-threatening disease. Therefore it was not included in the category 'fear for stigma'. Fear to be diagnosed positive could instead be considered a proxy for perception of risk, since persons mentioning this as a barrier for testing were 5 times more likely to have felt at risk (*P *= 0.001) and 4 times more likely to have expected the result to be positive (*P *= 0.001). Of the individuals that presented late and mentioned not-having-symptoms-yet as a barrier, 9 (69%) had felt at risk, indicating that feeling healthy might prevent people from converting their perception of risk into the act of HIV-testing. Other studies found that on average individuals who felt at risk of HIV infection wait for a year before testing, most needing a trigger [40] such as feeling ill which was the second most important reason to get tested in one of these studies [30].

As in other studies, we found heterosexual men more likely to present late than women and homosexual men [10,26-28,30]. Nevertheless, the proportion of women and homosexual men found to present late was still 30%. Almost one third of the women and the bi- and heterosexual men were tested for HIV as part of screening, whereas this proportion was only 9% among homosexuals. Higher utilisation rates of health services and regular HIV screening during antenatal care could explain the lower likelihood of women presenting late to diagnosis [26,28,33], however, in our study only 5/25 early presenting women were diagnosed during antenatal screening. In accordance with an Italian study showing that women tested more because of sexual contact with an HIV-infected person [26], a quarter of the women (and homosexual men) in our study went for an HIV test because their partner was HIV-infected or had signs/symptoms of possible infection, while among hetero- and bisexual men, these proportions were respectively 6% and 8% (data not shown). In our study, homosexual men were wealthier, had enjoyed higher education and had higher knowledge of HIV/AIDS and of the PNSIDA than women and other men. Since these factors were related to early testing in other studies this could explain why homosexuals were less likely to present late [26,27]. It has also been shown that those homosexuals who are integrated into the gay community are more likely to test for HIV [41]. Even though our sample was not sufficiently large to analyse each subgroup separately, our findings indicate that women, bi-, homo-, and heterosexual men may have different pathways to testing and different factors related to late presentation.

## Conclusion

As observed elsewhere [12,19] the impact of ART on the prognosis of HIV-infected individuals has not substantially influenced people's behaviours and beliefs towards HIV testing in Venezuela. Although Venezuela offers free diagnosis and treatment as part of its National HIV Program, an important proportion of individuals present late for HIV diagnosis. Older age, male heterosexuality, low education, low socio-economic status, lack of perceived risk, barriers related to lack of knowledge and lack of awareness of both HIV and the Program itself were important determinants in this delay. Our study has given indications for areas of interest that should be explored further using more in-depth qualitative studies in order to determine what role the different components play in HIV-testing behaviours. Nevertheless, our study shows that even in the frame of free HIV control programs efforts must still be directed to increase awareness of HIV/AIDS and on the availability of the services offered by the HIV Program. Moreover, the identification of specific factors associated with delay in HIV-diagnosis per subgroup, women, bi-, homo-, and heterosexual men, will be useful in the development of targeted public health interventions increasing the likelihood of early diagnosis, and therefore, of the prognosis of people living with HIV/AIDS in Venezuela and elsewhere.

## Methods

### Study design and site

We performed a cross-sectional survey between May and October 2006 at the outpatient Centre of Integral Attention for STI and HIV/AIDS (CAI) in Valencia, to identify factors influencing delay in HIV-diagnosis using a case-case comparison. The CAI is the reference centre for the PNSIDA in Carabobo State. This State has a population of 2 million inhabitants of which 70% live in the metropolitan area of Valencia, the state capital [20]. The region is served by several public and private hospitals of various levels and has a reported HIV-incidence of 12.24/100,000 [42] and an AIDS-related mortality of 4.76/100.000 [43]. Besides the CAI, two tertiary level hospitals located in Valencia, are also part of the PNSIDA. Patients that are admitted to tertiary hospitals and diagnosed with HIV are reported to CAI. Patients with confirmed HIV-diagnosis (Western Blot) are included in the PNSIDA and notified to the regional Ministry of Health (INSALUD) through a National HIV Notification Form including epidemiological and clinical data. Risk factors and further clinical signs are recorded in the patients' medical files.

### Study population

The study population consisted of all individuals newly diagnosed with HIV-infection at CAI between May 2005 and October 2006. We chose recently diagnosed patients in order to minimise recall bias at the moment of interview (see below). Eligible patients were assigned a unique identification number to ensure anonymity of the collected data.

### Data collection

A structured questionnaire was developed to ascertain socio-economic details and factors related to testing behaviour. Most questions referred to the time before or at diagnosis. The questionnaire contained pre-coded as well as open questions, and was developed in English, translated in to Spanish and pre-tested and adapted during a pilot study. A social worker specialised in HIV/AIDS counselling and a medical doctor from CAI assisted in the development of the questionnaire and were trained to perform the interviews. Eligible individuals attending CAI were interviewed after being explained the purpose of the study and obtaining oral informed consent. Questionnaires were double-checked for consistency and entered into EPI-Info (version 6.04). Demographic and behavioural characteristics and medical details were collected from the patients' medical files.

### Measures

Late presentation at diagnosis, the outcome variable, was defined as patients classified at diagnosis with HIV disease-stage B or C according to the 1993 Centers for Disease Control and Prevention (CDC) classification compared to patients diagnosed in disease-stage A ('early presentation') [21]. This definition was chosen to avoid ascertainment bias when using CD4 counts or AIDS to define late presentation, since around 60% of individuals do not have a CD4 count up to at least 3 months after HIV diagnosis [4]. Moreover, differential distribution of individuals without CD4 counts introduces further bias as the majority of these correspond to disease stage A.

#### Demographic characteristic

Demographic characteristic: age, marital status, number of children, level of education, and occupation determined at HIV-diagnosis were collected from the patients' medical files. Proxy measures of socio-economic status (SES) were collected through interview: area of residence (rural, urban), characteristics of residence (availability of sanitary services and electricity, ownership, number of bedrooms), monthly household income and number of people living in the household. The CAI and the residences of the studied individuals were geo-located using a handheld Global Positioning System (GPS) (Garmin GPS 12, Software 4.51, Garmin Corp.) and downloaded into a digital map of Venezuela using Mapsource™ (Garmin Corp). ESRI ArcMap 9.1 was used to calculate straight-line distances from the subjects' residences to the CAI.

#### Behavioural characteristics

Behavioural characteristics were collected from the patients' medical files: sexual orientation, age at first sexual contact, condom use, having a steady partner and HIV-status, alcohol use and (injecting) drug abuse. Lifetime total number of sexual partners and casual sexual partners, perceived faithfulness of their steady partner, sexual contact with commercial sex workers and previous occurrence of STIs was recorded during interview.

#### Knowledge of HIV/AIDS

Knowledge of HIV/AIDS before HIV-diagnosis was assessed during the interview by the following: having ever heard of HIV/AIDS and how; a 15-item question about HIV-transmission; and six true-or-false statements about HIV/AIDS. A HIV-transmission-knowledge score was calculated assigning points for each correct mode of transmission (range 0–15). Knowledge of existence, availability and prices of HIV-tests as well as ART was assessed. A PNSIDA awareness score was calculated adding one point if there was awareness of: existence of test; free testing; existence of treatment; free treatment; treatment availability in Carabobo, in hospitals and at CAI. A total-HIV/AIDS-knowledge score (maximum of 28) was calculated by adding all scores (Table 3).

#### Risk perception and barriers to HIV-testing

Perception of risk of HIV-infection and health-seeking behaviour before HIV-diagnosis was assessed during the interview, as well as the reasons why the subject did or did not feel at risk. Regardless of their risk perception, all subjects were asked whether they had perceived any barriers to HIV-testing and a list of possible barriers was probed. People could mention more than one barrier. Following Awad et al. (2004), answers were classified in three main categories: i) fear for HIV-related stigma, consisting of "fear of loosing partner, friends and family, children or employment" and "fear of being rejected;" ii) fear for confidentiality at testing site, consisting of "fear that the test would not be held confidential," "expecting the results not to be correct," "worries about the attitude of the personnel at the testing site" and "fear of being seen at the testing site"; and iii) logistical constrains, consisting of "no time to go," "inconvenient location of testing site," "no money for transport costs," "not able to afford the test or treatment." Other possible barriers not belonging to these main categories were "not feeling at risk," "not having symptoms," "not wanting to know their HIV status," "fear to be diagnosed positive" and "not knowing where to go for HIV-test [44]." Furthermore, time and costs of travel to the CAI were asked.

#### Facilitators for testing

The reason why people took an HIV-test were noted during the interview and grouped into categories as follows: i) Screening: blood bank, antenatal and pre-surgery screening and screening as part of health certification; ii) Health-related reasons: referral by a health centre, the respondent or partner showing HIV-related symptoms and having HIV-infected partners or children; iii) Own initiative: testing because of curiosity, feeling at risk of HIV-infection, having had STIs, many sexual partners, unprotected sex, an unfaithful partner, or testing on advice of partner, family or friends.

### Analyses

Weather the interviewed sample was representative was examined by comparing the data obtained from the patients' medical files of interviewed and non-interviewed subjects. Proportions were compared using *x*^2 ^test, or Fisher's exact test when appropriate, and Student t-test to compare means. To obtain a relative measure of SES, a weighted scoring of occupation and proxy measures of SES was developed using principal component analysis (PCA) [45,46], so that each individual was classified into high or low relative wealth. Logistic regression was used to obtain crude and adjusted (for age group [<30, ≥ 30 years] and sex) odds ratios (OR) for socio-demographic, socio-economic and behavioural characteristics, HIV knowledge, risk perception, and barriers and facilitators for testing. Significance was determined at the 5% level (*P*-value<0.05) using Wald *P*-values. The Mantel-Haenszel score test examined trends in ordered categorical variables. For most of the factors related to risk perception, and barriers and facilitators for testing no adjustment was made for additional confounders as the aim was to describe the relative risk of factors that may be associated to late presentation rather than to isolate the specific effect of a particular variable. All other factors found to approach significance (p < 0.2) after adjusting for age-group and sex were fitted in a logistic regression model and adjusted for confounders. Effect modification of different variables was analysed and resulting models compared by likelihood ratio test. A final model included the factors remaining significant after adjusting for all other factors in the model, and the factors which substantially changed the OR of other variables. All statistical analyses were conducted using SPSS software (version 13.0.1; SPSS) and Stata software (version 8.0; Stata). Ethics clearance for the study was obtained from the ethics commission of INSALUD.

## Abbreviations

AIDS: Acquired Immune Deficiency Syndrome; ART: Antiretroviral therapy; CAI: Centre of Integral Attention for STI and HIV/AIDS; CDC: Centers for Disease Control and Prevention; CI: Confidence interval; GPS: Global positioning system; HIV: Human Immunodeficiency Virus; INSALUD: Carabobo State Ministry of Health; OR: Odds ratio; PLWHA: People living with HIV/AIDS; PNSIDA: Venezuelan National HIV/AIDS Program; PCA: Principal component analysis; SES: Socio-economic status; STI: Sexually transmitted infections.

## Competing interests

The authors declare that they have no competing interests.

## Authors' contributions

MAB participated in the design of the study and the questionnaire, coordinated and carried out data collection, performed the statistical analysis, interpreted the data and drafted the manuscript. MM participated in the design of the study and the questionnaire, assisted in the coordination of the study, carried out interviews and data collection, and interpreted the data. MZ participated in the design of the study and the questionnaire and carried out interviews. GM and MC participated in the design of the study and the questionnaire and assisted in the coordination of the study. FGW assisted in the coordination of the study and carried out parts of the data collection from the medical files. CL participated in the design of the study and the questionnaire and helped to collect the data and to draft the manuscript. RNI participated in the design and coordination of the study and critically revised the manuscript. AT conceived and designed the study, participated in the coordination, data collection, analysis and interpretation of the data, and the drafting and critical revision of the manuscript. All authors revised the manuscript critically and read and approved the final version.
